# Encoding of Oscillations by Axonal Bursts in Inferior Olive Neurons

**DOI:** 10.1016/j.neuron.2009.03.023

**Published:** 2009-05-14

**Authors:** Alexandre Mathy, Sara S.N. Ho, Jenny T. Davie, Ian C. Duguid, Beverley A. Clark, Michael Häusser

**Affiliations:** 1 Wolfson Institute for Biomedical Research and Department of Neuroscience, Physiology and Pharmacology, University College London, Gower Street, London WC1E 6BT, UK

**Keywords:** SYSNEURO, SIGNALING

## Abstract

Inferior olive neurons regulate plasticity and timing in the cerebellar cortex via the climbing fiber pathway, but direct characterization of the output of this nucleus has remained elusive. We show that single somatic action potentials in olivary neurons are translated into a burst of axonal spikes. The number of spikes in the burst depends on the phase of subthreshold oscillations and, therefore, encodes the state of the olivary network. These bursts can be successfully transmitted to the cerebellar cortex in vivo, having a significant impact on Purkinje cells. They enhance dendritic spikes, modulate the complex spike pattern, and promote short-term and long-term plasticity at parallel fiber synapses in a manner dependent on the number of spikes in the burst. Our results challenge the view that the climbing fiber conveys an all-or-none signal to the cerebellar cortex and help to link learning and timing theories of olivocerebellar function.

## Introduction

The climbing fibers are formed by the axons of the neurons in the inferior olive and are a major route for sensory information flow into the cerebellar cortex. It is therefore crucial to understand what information is conveyed by the climbing fibers and how it influences cerebellar processing. There are two major theories of climbing fiber function. According to one view, subthreshold membrane potential oscillations, which are synchronized across olivary neurons by gap junctions (Lampl and Yarom, 1993, 1997; Llinas et al., 1974; Llinas and Yarom, 1986), provide a timing signal regulating the output of the downstream Purkinje cells (Welsh et al., 1995; Yarom and Cohen, 2002). An alternative view proposes that the climbing fiber is involved in motor learning in the cerebellar cortex, acting as an error signal to trigger associative synaptic plasticity at parallel fiber synapses (Albus, 1971; Ito, 2001; Marr, 1969). While these two views are not mutually exclusive, it has proven difficult to integrate them (Mauk et al., 2000).

To resolve this debate, we need a better understanding of how somatodendritic signals in olivary neurons are converted into spiking patterns in olivary axons and how this is related to the state of the olivary network. Furthermore, it is also necessary to understand the effect of different patterns of axonal spiking on the downstream Purkinje cells. Olivary neurons have a remarkable electrophysiological signature. They exhibit prominent subthreshold membrane potential oscillations driven by dendritic calcium conductances (Lampl and Yarom, 1993, 1997; Llinas and Yarom, 1986). These oscillations are synchronized across neighboring neurons within the olivary network by gap junctions (Devor and Yarom, 2002b; Leznik and Llinas, 2005), which can be uncoupled to generate dynamic cell assemblies (Llinas and Sasaki, 1989; Welsh et al., 1995). When activated by depolarization or synaptic input, olivary neurons generate a characteristic response sequence (Llinas and Yarom, 1981a, 1981b) consisting of an action potential immediately followed by a prolonged afterdepolarization (ADP) lasting up to 10 ms and terminated by a pronounced afterhyperpolarization (AHP). It is unclear how this sequence of events relates to patterns of output in the axon and, importantly, how the subthreshold oscillations affect this process. This is particularly important in light of the possibility that olivary axons may transmit bursts, which was originally proposed in the 1960s (Armstrong and Rawson, 1979; Crill, 1970; Eccles et al., 1966) and has recently received renewed attention (Maruta et al., 2007). To address these issues, we have made direct patch-clamp recordings from the axons of olivary neurons in rat brainstem slices, simultaneously with somatic patch-clamp recordings. Our findings provide direct evidence for axonal bursts and their modulation, leading to new insights into the information conveyed by the olivary axon about the state of the olivary network. We further demonstrate using a combination of in vitro and in vivo recordings from Purkinje cells that this information can be relayed to the cerebellar cortex, where it enhances Purkinje cell output and both short-term and long-term plasticity at parallel fiber synapses.

## Results

### Olivary Axons Generate Bursts

**Figure 1:**
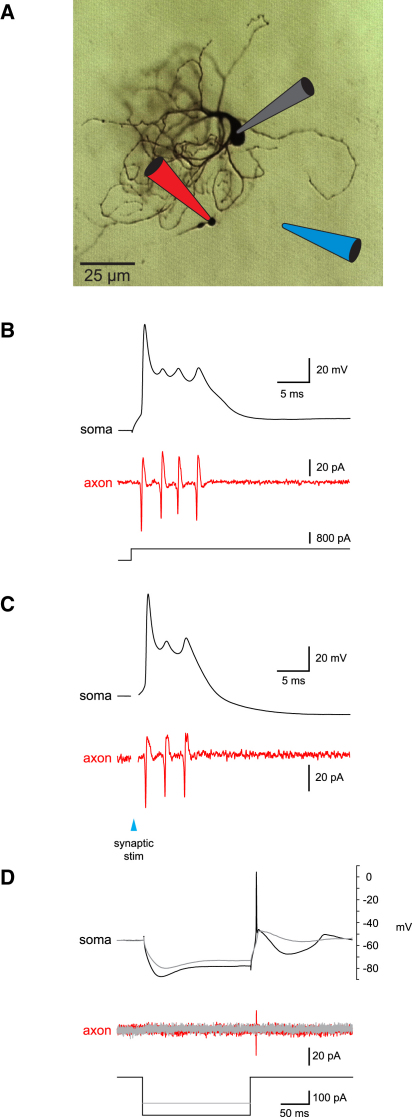
Axonal Recordings from Olivary Neurons (A) Recording configuration: whole-cell recording from the soma of an olivary neuron and cell-attached recording from an axonal bleb. A stimulation electrode was placed in the white matter. (B) Simultaneous somatic and axonal recording (130 μm from the soma) from an olivary neuron illustrating the response to somatic current injection. The small wavelets on the somatic ADP (top black trace) are reflected as full spikes in the axon (red trace). (C) Response to synaptic stimulation (same cell as in [B]). (D) Rebound spike upon release from hyperpolarization by a negative current pulse; a subthreshold (gray) and a suprathreshold response are shown (axonal recording 105 μm from soma).

Whole-cell somatic patch-clamp recordings were made from principal neurons in slices of the rat inferior olive under direct visual control. Principal neurons were identified by their large somata and characteristic electrophysiological properties (in particular the presence of a spike-ADP-AHP sequence upon depolarization; Crill, 1970; Llinas et al., 1974; Llinas and Yarom, 1981a, 1981b), confirmed by biocytin staining (Figure 1A). Axons were visualized by filling neurons via the patch pipette with Alexa Fluor 488, and simultaneous cell-attached axonal recordings were made from the cut ends of the axons using a second patch pipette at distances of up to 275 μm from the soma (n = 32).

Action potentials triggered by depolarizing current pulses (Figure 1B) or by single EPSPs evoked by white matter stimulation (Figure 1C) were followed by a pronounced “shoulder,” or afterdepolarization (ADP), on which was superimposed one to six small (<10 mV) wavelets at high frequency. Our simultaneous axonal recordings revealed that each wavelet corresponded to a spike in the axon with amplitude and kinetics comparable (and often identical) to the axonal counterpart of the primary sodium action potential. Thus, an action potential triggered by depolarizing current or a single synaptic stimulus was associated with a burst of axonal spikes (on average 2.2 ± 0.16 spikes, range 1–6 spikes, at an instantaneous frequency of 273 ± 9 Hz, range 127–476 Hz; n = 23 cells). In contrast, a rebound action potential triggered following the termination of hyperpolarizing current pulses lacked the pronounced ADP and was only associated with a single axonal spike (Figure 1D). Subthreshold low-threshold calcium spikes and oscillations (Llinas and Yarom, 1981a) on their own were not associated with axonal spikes. These findings demonstrate that the characteristic ADP of olivary neurons is converted into a spike burst in the axon and that the wavelets superimposed on the ADP correspond to axonal spikes.

### Bursts Are Initiated in the Olivary Axons

**Figure 2:**
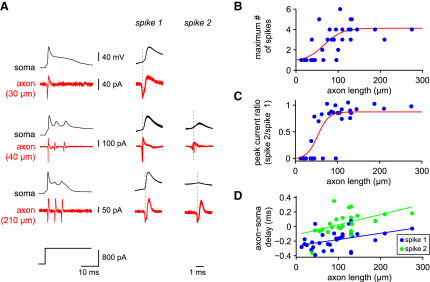
Spike Initiation in the Olive (A) Left column: somatic and axonal traces from cells with different axonal lengths. Cells were depolarized by a somatic current injection. The second and third columns show an overlay of five axonal and somatic traces from these cells at a faster time base for the first (“spike 1”) and second (“spike 2”) spike in the burst, respectively. (B) Maximum number of spikes in the bursts recorded from the neurons as a function of axon length. A sigmoidal fit is shown (red). (C) The ratio of the peak axonal current amplitude for the second spike to the first spike in the burst as a function of the length of the axon. A sigmoidal fit is shown (red). (D) Delay between the axonal and somatic spike as a function of axon length for the first and second spike in an olivary burst. The lines of best fit for each spike are plotted in blue and green, respectively.

To determine the site of origin of the bursts in the olivary axons, we examined our simultaneous somatic whole-cell recordings and axonal cell-attached recordings, with axonal morphology determined by subsequent biocytin processing and reconstruction. We found that the number of axonal spikes depended on the length of the axon spared by the slicing procedure. When axon length was shorter than 50 μm, most cells did not exhibit any secondary spikes or corresponding wavelets in the somatic ADP, despite the primary action potential being clearly present both at the soma and in the axon (Figure 2A; n = 6/8 cells). Only with an axon length of at least 100 μm were multiple spikes consistently observed in the axon, with a saturating relationship between axon length and maximum number of axonal spikes (Figure 2B; half maximum at 67 μm). A comparable relationship was observed for the mean number of axonal spikes (see Figure S1 available online). Similarly, the amplitude of the secondary spikes relative to the primary spike depended on the length of axon (Figure 2C; half maximum at 53 μm), with the amplitude of primary and secondary spikes becoming comparable at axon lengths of at least 100 μm (statistically indistinguishable results were obtained with spikes triggered by current injection and synaptic stimulation; data not shown, p > 0.05). In contrast, the somatic input resistance did not depend on the length of the axon (mean 97 ± 7.5 MΩ; *r* = −0.088, p > 0.05). Taken together, these findings suggest that the secondary spikelets in the axon, corresponding to the wavelets in the ADP, are initiated in the axon. To confirm this, we estimated the latency between the axonal and somatic spikes, by measuring the time between the peak of the axonal spike and the peak of the dV/dt of the somatic action potential (Figure S2; Clark et al., 2005; Meeks and Mennerick, 2007). We found that for both the primary and secondary spikes, the axonal spike clearly preceded the somatic spike for most axonal recording sites less than 100 μm from the soma (Figure 2D), indicating that spikes are initiated in the axon relatively close to the soma.

### Whole-Cell Recordings from Olivary Axons

**Figure 3:**
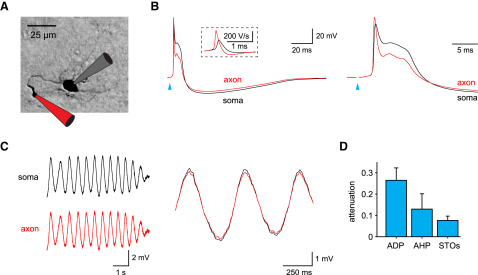
Whole-Cell Recordings from Olivary Axons (A) Recording configuration: simultaneous whole-cell recording from the soma and axon of an olivary neuron (45 μm from the soma). (B) Somatic and axonal voltage responses of the cell in (A) to synaptic stimulation (at the arrowhead). Left and right panels show the same data at different timescales. Note the marked attenuation of the ADP in the axon. Inset: dV/dt of the somatic and axonal traces. The peak axonal dV/dt occurs before that of the soma. Right: same data at a faster time base. (C) Left: spontaneous subthreshold oscillation recorded in the soma and axon of another cell (axonal recording 35 μm from the soma). Right: same traces smoothed and overlaid at a faster time base shows the axonal attenuation of the oscillation. (D) Axonal attenuation of the ADP and AHP (mean ± SEM, n = 5 cells) and the spontaneous subthreshold oscillations (n = 2 cells). Mean axonal length for this data = 38 ± 4 μm.

We succeeded in making simultaneous whole-cell recordings from the soma and cut end of the axon of the same neuron when the axon was cut within 60 μm of the soma, the approximate distance at which myelin normally begins to ensheath the olivary neuron axons (de Zeeuw et al., 1990). While these axons did not usually exhibit secondary spikes, consistent with the results shown above (Figure 2B), these recordings nevertheless allowed us to investigate initiation and propagation of subthreshold and suprathreshold potentials in the axon. The primary spike was consistently larger in the axon than at the soma and occurred first at the axonal recording site (Figure 3B; n = 5), in agreement with an axonal initiation site. In contrast, the ADP was larger at the soma and strongly attenuated in the axon. The AHP was also larger at the soma but suffered much less attenuation in the axon than the ADP (Figures 3B and 3D). Spontaneous subthreshold oscillations (STO), a hallmark of olivary neurons (Chorev et al., 2007; Khosrovani et al., 2007; Llinas and Yarom, 1986), were larger at the soma but showed little attenuation in the axon (Figures 3C and 3D), probably due to their low frequency (1–10 Hz). These findings indicate that, while spikes are initiated in the axon, the ADP, the AHP and the subthreshold oscillations most likely originate from the somatodendritic region.

### Phase Dependence of Olivary Bursts during Subthreshold Oscillations

**Figure 4:**
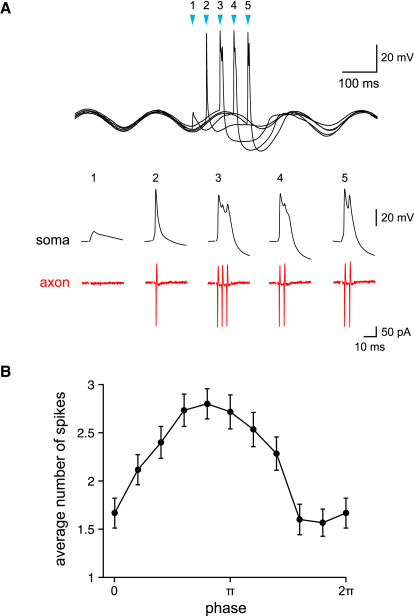
Modulation of Olivary Output by Subthreshold Oscillations (A) Top panel: overlay of five somatic responses of an olivary neuron to synaptic stimulation during subthreshold oscillations generated by injection of a sinusoidal (5 Hz) current. EPSPs were triggered by single synaptic stimuli (arrowheads), timed to occur at different phases of the oscillation. Lower panel: same somatic responses on an expanded timescale and, below, the simultaneously recorded axonal spikes. (B) Average number of spikes evoked (mean ± SEM, pooled data, n = 14 cells) as a function of the phase of synaptic stimulation.

The subthreshold membrane potential oscillations exhibited by olivary neurons range from 1 to 10 Hz in frequency and from 1 to 20 mV in amplitude (Chorev et al., 2007; Khosrovani et al., 2007; Llinas and Yarom, 1986). To determine if oscillations can modulate synaptically triggered axonal bursts, we mimicked subthreshold oscillations by injecting a sinusoidal current into the soma, with a frequency of 5 Hz and producing a mean peak-to-peak amplitude of 9.6 ± 0.6 mV. Spikes were initiated by EPSPs activated by synaptic stimulation timed at different phases of the oscillation (n = 14 cells). The number of spikes in the axonal burst was modulated in phase with the oscillation (Figure 4B). For the example shown in Figure 4A, the mean number of spikes triggered by synaptic stimulation in the absence of oscillations was 2.07 ± 0.03, with a low coefficient of variation (CV) of 0.17. During oscillations, synaptic input triggered from zero to three spikes (mean 2.40 ± 0.17, coefficient of variation 0.49), with the number of spikes depending on the timing of the EPSP relative to oscillatory phase. This indicates that the phase of the oscillation can regulate the output of olivary neurons.

In olivary neurons that exhibited spontaneous oscillations (Figure S3), we investigated whether the spike output was affected by the phase of the oscillation. The mean oscillation frequency was 5.6 ± 0.65 Hz (n = 7) and the peak-to-peak amplitude was 6.6 ± 0.94 mV. Because synaptic stimulation abolishes oscillations in olivary slice preparations (Lampl and Yarom, 1993), we injected short current pulses (2 ms; 300–1800 pA) at different phases of the oscillation (Figure S3B) to trigger spikes and characterized the axonal spike output by counting the number of wavelets on the ADP. Confirming our results with injected oscillations, we found that the spike output varied with the phase of the oscillation (Figure S3C).

### Reliability and Precision of Axonal Transmission of Bursts

**Figure 5:**
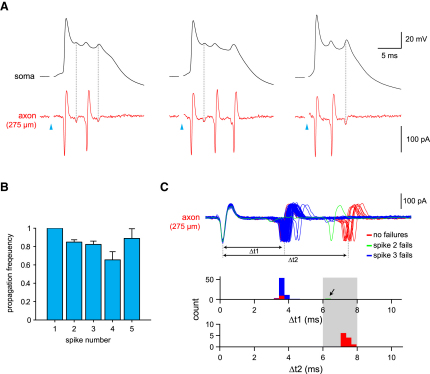
Imperfect Axonal Transmission of Olivary Bursts (A) Three responses of a cell to synaptic input (triangles) showing failures of axonal spike propagation at 275 μm from the soma. Dotted lines indicate somatic wavelets (top traces) for which a corresponding full axonal spike was absent (bottom traces), indicating a propagation failure. (B) Propagation probability for each spike in a burst (mean ± SEM, n = 10 cells). (C) Top: overlay of 85 consecutive axonal traces of burst responses in the same neuron as in (A). Blue and green traces show failures of the second and third spike, respectively. Bottom: corresponding histograms for these data of time intervals between the first and second axonal spike (top histogram) and the first and third axonal spike (bottom histogram).

A 1:1 relationship was typically observed between wavelets on the somatic ADP and axonal spikes. However, in the majority of axonal recordings made at >125 μm from the soma (7/10 cells), occasional failures of propagation of secondary spikes were observed (i.e., cases where a somatic wavelet did not coincide with a detectable spike at the axonal recording site; Figure 5A). This probably occurred due to failure of orthodromic propagation of spikes initiated in the more proximal axon. Intriguingly, all axonal spikes always had a somatic counterpart, indicating that propagation of the axonally generated spike back to the soma always results in a wavelet. We next quantified the reliability of propagation of individual spikes within the burst. Propagation of the primary spike was 100% reliable. Spikes in the middle of the burst—i.e., the third and fourth spike—were most susceptible to propagation failure. The second, third, fourth, and fifth spikes in the burst exhibited propagation probabilities of 0.85 ± 0.03, 0.82 ± 0.04, and 0.66 ± 0.09, and 0.89 ± 0.10, respectively (Figure 5B), indicating that propagation is relatively reliable—despite occasional failures—across the burst.

In addition to the relatively reliable axonal propagation of spikes, the timing of individual spikes within the burst was remarkably consistent from trial to trial. As shown in Figure 5C, the interspike interval between the first and second spikes in a burst was highly reliable, with a CV of only 0.12 ± 0.02 (n = 23 cells). The succeeding intervals were similarly reliable (second interval CV: 0.20 ± 0.03; third interval CV: 0.18 ± 0.06). The first ISI in the burst was 3.98 ± 0.19 ms for a two-burst and 3.53 ± 0.51 for a three-burst. These findings indicate that the timing of spikes within a burst in the olivary axon is highly stereotyped (assured in part by reliable transmission of each spike), with only the number of spikes varying.

### Transmission of Axonal Bursts In Vivo

**Figure 6:**
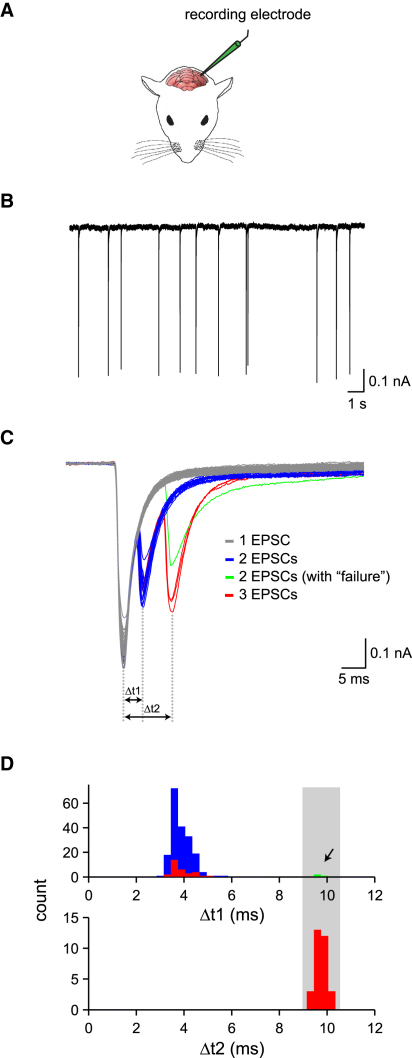
In Vivo Transmission of Olivary Bursts (A) Recording configuration for in vivo whole-cell voltage-clamp recordings from Purkinje cells. (B) Spontaneous all-or-none CF EPSCs recorded from a Purkinje cell in vivo (holding potential −90 mV). (C) Overlay of individual EPSCs from the same recording shown in (B). The events contain one, two, or three clearly separated peaks (gray, red, and blue traces, respectively). In the green trace, a putative failure of a presynaptic spike occurs between the two EPSCs. (D) Corresponding histograms of time intervals between the first and second EPSC (top histogram) and the first and third EPSC (bottom histogram) for the cell shown in (B). Note that the second peak (green, arrow) in the top histogram coincides with the range of the bottom histogram (gray shaded region).

Can bursts of action potentials in the axons of the inferior olive neurons be faithfully propagated to their downstream targets, the Purkinje cells in the cerebellar cortex, under physiological conditions? To address this question, we recorded climbing fiber excitatory postsynaptic currents (CF EPSCs) (Konnerth et al., 1990; Llano et al., 1991; Perkel et al., 1990) from Purkinje cells in vivo (Figure 6A). Given that the axonal bursts in olivary neurons have highly reproducible timing (Figure 5), if they are reliably transmitted to their synapses with Purkinje cells, then bursts of CF EPSCs with similar intervals and timing to the axonal bursts should be observed. To optimize the recording of CF EPSCs, we used an internal solution containing QX314 and held Purkinje cells at hyperpolarized potentials (−70 mV to −100 mV) in order to prevent unclamped spikes and regenerative responses (Llano et al., 1991).

CF EPSCs were identified based on their characteristic large-amplitude and all-or-none nature (Figure 6B), as reported in vitro (Konnerth et al., 1990; Llano et al., 1991; Perkel et al., 1990). CF EPSCs had a relatively low mean frequency (on average 0.63 ± 0.03 Hz; range: 0.24–1.85 Hz), comparable to the rate of spontaneous complex spikes recorded in vivo (Armstrong and Rawson, 1979; Lang et al., 1999; Loewenstein et al., 2005). In approximately half of the Purkinje cells recorded (n = 16/33), the initial CF EPSC was followed by one or more additional EPSCs in a burst-like pattern (at 222 ± 46 Hz; range: 91–357 Hz; first ISI: 4.07 ± 0.094 ms, second ISI: 4.17 ± 0.19 ms); bursts comprised up to four individual CF EPSCs in some Purkinje cells (Figure S4). The frequency of occurrence of bursts in a given Purkinje cell was voltage independent when varying holding potential between −70 mV and −90 mV (n = 6; p = 0.67), confirming their presynaptic origin. By overlaying multiple spontaneous CF EPSC bursts from the same cell, the timing of individual EPSCs within the burst was observed to be very reproducible (Figures 6C and S4). In particular, the timing of the second EPSC in the burst was highly precise (CV = 0.18 ± 0.05; n = 16 cells), very similar to the timing of the second spike in a burst in an olivary axon (cf. Figure 5C; p > 0.1), confirming that the axonal burst in olivary neurons can be reliably and precisely transmitted to Purkinje cells. The frequency of double- and triple-peak CF EPSCs was 15.9% ± 1.6% (range: 0.1%–80%; n = 16) and 4.2% ± 0.6% (range: 0%–35%; n = 6), respectively. Remarkably, in some cells we were able to observe the putative postsynaptic counterpart of the occasional failures of spike propagation observed in our axonal recordings (Figure 5). In these rare cases, the second EPSC occurred at precisely the time of the third EPSC in a burst (Figure 6C), suggesting that the second spike in an olivary burst failed to be transmitted. These findings demonstrate that olivary bursts are transmitted via the climbing fibers to their target Purkinje cells in the cerebellar cortex in vivo and that occasional failures of spike transmission can be observed.

### Climbing Fiber Bursts Shape the Complex Spike Response in Purkinje Cells

**Figure 7:**
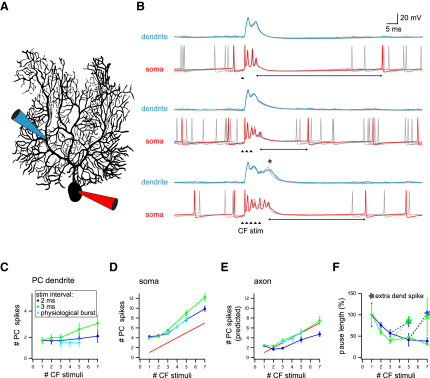
Response of Purkinje Cells to Bursts of CF Input (A) Recording configuration for simultaneous whole-cell somatic and dendritic recordings from Purkinje cells in acute slices. (B) Example dendritic (upper traces, recorded 85 μm from the soma) and somatic (lower traces) Purkinje cell responses to one, three, and five CF stimuli at 2 ms intervals (black triangles). Several superimposed traces (gray); single examples shown in blue (dendritic recording) and red (somatic recording). Dendritic and somatic data are shown with different timescales. Blue arrows, post-complex spike pause duration; asterisk, additional dendritic calcium spike. (C) Mean (±SEM) number of dendritic spikes (n = 3 cells) in response to CF burst stimulation. Stimuli were given at intervals of 2 ms (dark blue markers), 3 ms (green markers), or patterns mimicking recorded olivary axon bursts (light blue markers). (D) Mean (±SEM, n = 10) number of somatic spikes in response to CF burst stimulation, protocol as in (B). Red line marks unity, where Purkinje cell spike number is equal to number of CF stimuli. (E) Mean (±SEM, n = 7) number of CF burst-evoked spikes predicted to be propagated by the Purkinje cell axon (see Experimental Procedures). Stimulation protocol as in (B), red line as in (D). (F) Average (mean ± SEM) post-complex spike pause length following bursts of CF stimulation. Responses in which a burst of CF stimuli elicited additional dendritic spikes are averaged separately and marked with an asterisk.

To investigate the consequences of bursts in climbing fiber axons for postsynaptic Purkinje cells, we recorded from the soma, and in some cases, simultaneously from the dendrites, of Purkinje cells in slices during CF stimulation. Bursts of one to seven CF stimuli were delivered at interstimulus intervals of 2 or 3 ms (a range representative of interspike intervals recorded in olivary axons during bursts), or as “physiological” spike patterns recorded from olivary axons in slices. The number of dendritic spikes triggered by CF stimulation was related to the number of stimulations in a CF burst, with larger numbers of CF stimuli triggering additional dendritic spikes (Figures 7B and 7C). At the soma, the number of spikes in the somatic complex spike was dependent on the number of stimuli in the CF burst: there was a linear relationship between the number of CF stimuli and the number of somatic spikelets (Figures 7B and 7D). The duration of the complex spike was also linearly dependent on the number of CF stimuli across a range of interstimulus intervals (Figures S5A and S5B); at longer interstimulus intervals, CF bursts were more effective at triggering extra somatic spikes and produced a more prolonged complex spike. In summary, these results indicate that the number of spikes within the presynaptic CF burst can influence the number of spikes and duration of the resulting complex spike.

The effect of additional CF input also changed the pattern and timing of spikes within the complex spike. Rather than triggering a new somatic spike at a specific synaptic delay, an additional CF stimulus slightly advanced spikes within the ongoing complex spike, with the additional somatic spikes caused by bursty CF input appearing on the tail end of the complex spike. The first spike to be affected by an additional CF stimulus was advanced by 0.41 ± 0.11 ms (2 ms interstimulus interval) and 0.26 ± 0.11 ms (3 ms interstimulus interval) and was increased in precision; the SD of timing decreasing by 0.23 ± 0.12 ms and 0.11 ± 0.04 ms (2 and 3 ms interstimulus interval, respectively; Figures S5C and S5D).

Since somatic spikelets in the complex spike propagate relatively poorly down the Purkinje cell axon, we used spikelet height and interspikelet interval to predict which spikes would be successfully propagated down the axon (Monsivais et al., 2005; see also Khaliq and Raman, 2005). The predicted number of axonal spikes versus the number of CF stimuli, using physiological input patterns, was very close to the unity line (Figure 7E), producing a “one in, one out” input-output relationship for axonal CF spikes and Purkinje cell output spikes. Finally, we examined the relationship between the CF burst and the pause in firing following the complex spike. To a first approximation, increasing the number of spikes in the CF burst decreased the length of the post-complex spike pause, as expected from the stronger excitation at the soma. However, when further burst stimulation succeeded in evoking an additional dendritic calcium spike (e.g., lowest panel of Figure 7B), the duration of the pause increased (Figure 7F), presumably because the dendritic spike in turn lengthens the post-complex spike pause (Davie et al., 2008). In summary, bursts of CF input can be encoded by the firing patterns of the postsynaptic Purkinje neurons, both in terms of dendritic spikes and axonal output.

### Enhancement of Long-Term Depression by Climbing Fiber Bursts

**Figure 8:**
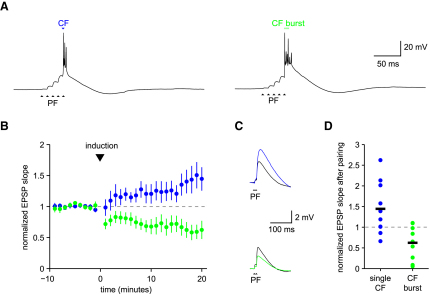
CF Bursts Enhance Long-Term Associative Plasticity of Purkinje Cells (A) Responses of two Purkinje cells to the two plasticity induction protocols used: a burst (100 Hz) of five parallel fiber stimuli in conjunction with a single CF stimulus (left) or a burst (400 Hz) of CF stimuli (right; 25 repetitions at 0.5 Hz). (B) Effect of the two induction protocols on PF synaptic strength (measured as normalized EPSP slope; mean ± SEM; CF burst: green symbols, n = 10; single CF stimulus: blue symbols; n = 9). (C) Black traces: PF-EPSPs averaged over a minute before induction in two cells. Induction was performed with the single CF and the CF burst protocol in the top and bottom cell, respectively. The colored traces show the EPSPs 20 min after induction. (D) Distribution of changes in PF synaptic strength 20 min after induction for all cells tested (CF burst: green symbols; single CF, blue symbols).

Given that CF bursts can regulate the number of dendritic spikes produced by CF input (Figure 7), we examined the consequences for short-term and long-term plasticity in Purkinje cells. Parallel fiber (PF) synapses are known to undergo long-term depression (LTD) when stimulated in conjunction with CF input (Ito, 2001; Wang et al., 2000). We therefore investigated whether the CF bursts had an effect on the induction of PF LTD. We used an induction protocol which paired five PF stimuli (at 100 Hz; (Chadderton et al., 2004) with either a single CF stimulus or a CF burst (five stimuli at 400 Hz; Figure 8A). We used only a small number of pairings (25) delivered every 2 s, which should normally provide only a relatively weak stimulus for plasticity (Jorntell and Hansel, 2006). Indeed, the induction protocol with a single CF stimulus produced potentiation in the control group (139% ± 20% compared to baseline; n = 10; p = 0.033). In contrast, when a single CF stimulus was replaced by a CF burst in the induction protocol, robust LTD was observed (63% ± 15% compared to baseline; n = 9; p < 0.01; Figures 8B–8D). These results demonstrate that CF bursts can enhance the probability of LTD induction using conjunctive PF-CF stimulation.

### Enhancement of Synaptically Evoked Suppression of Excitatory Synapses by Climbing Fiber Bursts

**Figure 9:**
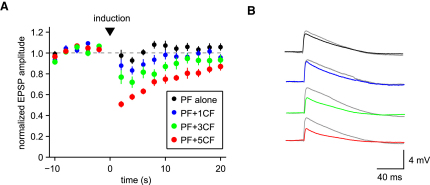
CF Bursts Enhance Short-Term Associative Plasticity at Parallel Fiber Synapses Synaptic suppression of excitation (SSE) of parallel fiber efficacy is also modulated by climbing fiber bursting. (A) Time course of PF-EPSP amplitude (mean ± SEM, n = 3) for induction with a burst of PF stimuli (black circles), or a PF burst in association with one, three, or five CF stimuli (blue, green, and red circles, respectively). (B) PF-EPSP traces averaged over five trials from a Purkinje cell. The gray traces are EPSPs 2 s before induction. The black, blue, green, and red traces are the EPSPs 2 s after induction with a burst of PF stimuli in association with zero, one, three, or five CF stimuli, respectively.

Pairing a short burst of PF stimuli with CF input can transiently and selectively depress the PF synapses, a form of associative short-term plasticity known as synaptically evoked suppression of excitatory synapses (SSE; Brenowitz and Regehr, 2005). We tested whether CF bursts could enhance SSE. For induction of SSE, we used a burst of four to six PF stimuli at 100 Hz followed by either a single CF stimulus or a burst of three or five CF stimuli at 400 Hz. When the CF stimulus was omitted, there was no change in synaptic efficacy at the PF synapses (2.5% ± 3% depression relative to baseline; n = 18 trials from 3 cells). When PF inputs were paired with a single CF stimulus, PF synapses were depressed by 12% ± 2.6% (n = 17 trials). When the PF stimulus was paired with a burst of three or five CF stimuli, the PF synapses were depressed by 23% ± 7% and 50% ± 2.4% (n = 17 trials), respectively. There was a significant correlation (*r* = 0.61, p < 0.001) between the number of CF stimuli in the induction protocol and the degree of PF depression (Figure 9). Together with the previous results, we conclude that CF bursts, driven by bursts in olivary axons, can enhance both short-term and long-term synaptic plasticity in postsynaptic Purkinje cells.

## Discussion

We have made direct recordings from the axons of inferior olive neurons, which form the climbing fibers of the cerebellum. We demonstrate that olivary axons can fire high-frequency bursts of action potentials that can be modulated by olivary oscillations and that are transmitted to the cerebellar cortex. This challenges the view of the climbing fiber as a low-frequency all-or-none signaling pathway. We further show that climbing fiber bursts have a dramatic effect on their postsynaptic targets, the Purkinje cells: they enhance dendritic spiking, modulate complex spike signaling, and promote short-term and long-term plasticity in cerebellar Purkinje cells in a manner that depends on the number of spikes in the input burst. Thus, bursts in axons of olivary neurons influence both short-term processing and memory storage in Purkinje cells. Our findings therefore provide a rich new perspective on the functional role of the climbing fiber system.

### Bursts in Olivary Axons

Our findings provide direct evidence that olivary axons can fire high-frequency, stereotyped bursts of action potentials in response to single synaptic potentials. Furthermore, we show that these bursts are transmitted to Purkinje cells in vivo via the climbing fibers. There has been previous indirect evidence that olivary axons may fire in bursts. Wavelets on the ADP of olivary neurons were observed in early intracellular recordings from the somata of olivary neurons both in vivo and in vitro, with impulse collision experiments suggesting that wavelets can propagate along the climbing fiber (Armstrong et al., 1968; Crill, 1970). Furthermore, several studies using sharp electrode recordings from Purkinje cells in vivo reported spontaneous trains of large EPSP-like events, which have been interpreted to arise from bursts in climbing fiber axons (Armstrong and Rawson, 1979; Eccles et al., 1966; Maruta et al., 2007). Our direct recordings of bursts in olivary axons, and our demonstration of their functional significance, should now confirm them as an important element in the operation of the olivocerebellar circuit.

### A Model of Spike Generation by Olivary Neurons

Our simultaneous axonal and somatic recordings provide new insights into the generation of the characteristic electrophysiological signature of olivary neurons. First, our results provide direct evidence that both the primary sodium spike and the secondary spikes originate in the olivary axon. Second, we demonstrate that the prominent subthreshold oscillations are smaller in the axon than at the soma, consistent with the idea that they originate in the dendrites and that the gap junctional coupling that transmits the oscillation between multiple olivary neurons is predominantly dendritic (Bal and McCormick, 1997; Llinas and Yarom, 1986). Finally, we show that the pronounced ADP is markedly attenuated in the axon, consistent with the hypothesis that the ADP is generated by high-voltage-activated calcium channels localized in the dendrites (Llinas and Yarom, 1981b).

Together, these results suggest a mechanism for burst generation in olivary neurons: the initial sodium action potential is initiated in the axon and backpropagates into the dendrites, activating the calcium channels that underlie the ADP. When the ADP reaches the soma, the plateau potential is close to 0 mV, a value at which somatic sodium channels will be mostly inactivated. We have demonstrated that the ADP is rapidly attenuated in the axon, such that when it reaches the action potential initiation site in the axon it provides sufficient depolarization to trigger repetitive axonal spikes, but insufficient depolarization to inactivate sodium channels. These spikes in turn propagate down the axon at the same time as backpropagating toward the soma, giving rise to small wavelets due to inactivation of the local sodium channels by the ADP. This picture can be compared to burst generation in cortical pyramidal neurons, where the backpropagation of the action potential into the dendrites activates calcium conductances that provide depolarization to the axon to generate bursting (Williams and Stuart, 1999). In contrast, in the olivary neurons, the ADP is large enough at the soma to inactivate somatic sodium channels to such a degree that full-amplitude spikes do not appear during the ADP, instead being replaced by small wavelets.

### Bursts Encode Oscillations

The prominent subthreshold oscillations in olivary neurons have been proposed to determine the timing of olivary output (Lampl and Yarom, 1993), but little attention has been paid to how the phase of the oscillation affects somatodendritic integration. By providing synaptic input at different times relative to subthreshold oscillations, we demonstrate that the number of spikes in the olivary burst depends on the phase of the oscillation. This is presumably due to differential availability or activation of dendritic calcium channels at different time points in the oscillation, which in turn sets the duration of the ADP and thus the number of spikes in the axonal burst (see above). In contrast, Crill (1970) has shown that the number of spikes in the response varies little with the strength of stimulation. Thus, olivary bursts provide a means of encoding the state of the olivary network in a more fine-grained manner than possible by a single all-or-none spike, but do not seem to convey information about the intensity of stimulus that triggered them.

### Transmission of Spikes by Olivary Axons

We have employed a dual strategy for investigating the transmission of bursts by olivary axons. First, we have made simultaneous somatic and axonal recordings in olivary slices. These recordings reveal that the transmission of olivary bursts down the axon is relatively reliable, with the entire burst normally being successfully transmitted. However, individual spikes in the olivary burst can fail with low probability. Second, in a complementary approach, we have made voltage-clamp recordings of climbing fiber EPSCs from Purkinje cells in vivo, which reveal that they can occur as bursts. These bursts are highly stereotyped, with interspike interval precision corresponding to that observed in olivary axons. This confirms that olivary bursts are transmitted along the full extent of the climbing fiber axons and successfully and reliably activate the downstream Purkinje cell targets. The fact that occasionally “skipped” EPSCs were observed in these recordings confirms that the occasional failures of axonal propagation observed in olivary slices also occur in vivo, and suggests that some modulation of axonal transmission may be possible. This overall picture contrasts with that observed in Purkinje cell axons, where propagation of complex spike bursts, which are of a similar frequency, is far less reliable (Khaliq and Raman, 2005; Monsivais et al., 2005). This strengthens the possibility that olivary bursts have functional significance and suggests that, compared to Purkinje cell axons, they express different types or densities of voltage-gated channels in order to improve the reliability of high-frequency axonal transmission.

### Impact of Olivary Axon Bursts on Purkinje Cell Dendritic Integration and Spike Output

For olivary bursts to have functional consequences, the postsynaptic partners of olivary neurons must be able to read out and modify their output based on the characteristics of the bursts. We have addressed this issue by making simultaneous somatic and dendritic recordings from Purkinje cells and activating the climbing fiber in different patterns. We demonstrate that bursts in the climbing fiber can promote generation of dendritic spikes in Purkinje cells. Furthermore, climbing fiber bursts can modify the number of spikelets in the complex spike and its duration, with both reflecting the number of spikes in the presynaptic burst. The resulting wide range of complex spike waveforms is consistent with the variability in complex spike patterns recorded in vivo at the somatic (Armstrong and Rawson, 1979; Campbell and Hesslow, 1986b; Gilbert, 1976; Mano, 1970) and axonal (Campbell and Hesslow, 1986a; Ito and Simpson, 1971) level. Finally, we demonstrate that the pause in simple spiking following the complex spike (Armstrong and Rawson, 1979; Bloedel and Roberts, 1971; Davie et al., 2008; Latham and Paul, 1970; Sato et al., 1992) also sensitively reflects both the number of spikes in the climbing fiber burst and its effect on dendritic activity. Thus, these results indicate that Purkinje cells can provide both dendritic and axosomatic readout of olivary activity. Using a model of axonal transmission of the complex spike (Monsivais et al., 2005; see also Khaliq and Raman, 2005), we predict that many of the additional spikelets in the somatic complex spike will be transmitted by the Purkinje cell axon, indicating that olivary bursts can drastically alter the output of the cerebellar cortex. The next step will be to determine how these changes in output are interpreted by the downstream synapses with the neurons of the deep cerebellar nuclei (De Zeeuw et al., 1997), which also receive input from climbing fiber collaterals and are thus also subject to the impact of climbing fiber bursts.

### Olivary Bursts Enhance Synaptic Plasticity in Purkinje Cells

Climbing fibers are known to play an important role in triggering plasticity in Purkinje cells (Ito, 2001). Our results indicate that bursts in the climbing fiber provide an enhanced stimulus for the induction of both associative short-term and long-term plasticity at parallel fiber synapses. This calls into question one of the fundamental assumptions of research into cerebellar plasticity, namely that the climbing fiber functions as an all-or-none trigger for associative plasticity. Rather, the sign and degree of plasticity is graded depending on the number of spikes in the climbing fiber burst, which in turn reflects the oscillatory state of the olive. This presumably in part reflects different levels of postsynaptic dendritic Ca^2+^ produced by different numbers of climbing fiber stimuli (Brenowitz and Regehr, 2005; Coesmans et al., 2004; Wang et al., 2000). While a few studies have used repetitive stimulation of climbing fibers for plasticity induction (Brenowitz and Regehr, 2005), none have explored the physiological range of climbing fiber burst frequencies shown here. Our findings indicate that, in order to understand physiological synaptic plasticity, burst stimulation of the climbing fiber should be considered when designing induction protocols for plasticity experiments. Furthermore, it will be important to investigate the impact of burst patterns for other kinds of plasticity involving the climbing fiber, such as CF LTD (Hansel and Linden, 2000).

### Implications for Cerebellar Function

There has been considerable controversy regarding whether the climbing fiber conveys a timing signal or an error signal that drives plasticity (Mauk et al., 2000). Subthreshold oscillations have been shown to play a crucial role in gating the response of olivary neurons to synaptic input, such that during the crest of an oscillation, the neuron is closer to threshold and hence a small stimulus will be more likely to cause spike output than at the trough of the cycle (Lampl and Yarom, 1993; Llinas and Yarom, 1986). This supports the idea that oscillations can act to synchronize olivary output (Devor, 2002; Long et al., 2002; Welsh et al., 1995; Yarom and Cohen, 2002). However, in vivo recordings indicate that the inferior olive can respond at very low thresholds with high probability and short latency to sensory stimulation (Gellman et al., 1985; Gellman et al., 1983) and that olivary neurons can fire at various times relative to the phase of the oscillation (Khosrovani et al., 2007). Furthermore, recordings of complex spike activity from Purkinje cells in vivo indicate that olivary spiking is at most weakly periodic (Chorev et al., 2007; Keating and Thach, 1995, 1997; Lang et al., 1999). Together these findings suggest that olivary spikes are not necessarily restricted to a specific phase of the oscillation, challenging the view that subthreshold oscillations are strict determinants of olivary timing.

We have shown that olivary neurons do not generate a unitary, all-or-none spike output. Rather, axonal output is in the form of a burst, with the number of spikes in the burst being graded, depending on the phase of the oscillation. This mechanism therefore allows olivary axons to convey information about the timing of the spike relative to the phase of the oscillation, even when the spike occurs out of phase. Thus, rather than serving to precisely time and synchronize olivary spikes (direct triggering of spikes via strong gap junctional coupling would provide a better candidate for a precise synchronization mechanism; see Devor and Yarom, 2002a; Long et al., 2002), subthreshold oscillations may function as a time-keeping device, assigning more saliency to in-phase stimuli. Thus, bursts in olivary axons serve to amplify any phase-dependence of olivary spiking in response to input, maximizing the signal-to-noise of in-phase transmission of information from the olive. These bursts are in turn read out by the downstream Purkinje cells, by enhancing dendritic spikes, axonal output, and synaptic plasticity. Our findings can therefore help to reconcile the competing theories of olivocerebellar function, in that olivary bursts provide a mechanism for oscillatory phase-dependent learning of parallel fiber input patterns in Purkinje cells, thereby linking the timing and learning aspects of motor function.

## Experimental Procedures

### Slice Preparation

Sagittal brain slices of the inferior olive (300 μm) and the cerebellar vermis (250 μm) were prepared from Sprague Dawley (P18–P25) rats in accordance with national and institutional guidelines. Rats were anesthetized with isoflurane and subsequently decapitated. The brain was removed and submerged in ice-cold artificial cerebrospinal fluid (ACSF) bubbled with carbogen (95% O_2_, 5% CO_2_). For inferior olive slices, the slicing ACSF contained (in mM) 250 sucrose, 25 NaHCO_3_, 10 glucose, 5 KCl, 1.25 NaH_2_PO_4_, 0.5 CaCl_2_, 3.5 MgCl_2_; and for cerebellar slices, the slicing ACSF contained (in mM) 125 NaCl, 25 NaHCO_3_, 25 glucose, 2.5 KCl, 1.25 NaH_2_PO_4_, 1 CaCl_2_, 4 MgCl_2_. The brain was cut parallel to the plane of slicing, and cyanoacrylate adhesive was used to fix the brain to the platform of a Leica VT-1000 vibratome. Slices were transferred to a holding chamber and incubated for 30 min at 34°C in standard ACSF containing (in mM) 125 NaCl, 25 NaHCO_3_, 25 glucose, 2.5 KCl, 1.25 NaH_2_PO_4_, 2 CaCl_2_, 1 MgCl_2_. Slices were then kept at room temperature until they were transferred to a recording chamber and continuously superfused with oxygenated standard ACSF (same composition as above, except olivary experiments were done with 5 mM KCl). All recordings were made at 34°C ± 1°C.

### Slice Electrophysiology

Patch pipettes were pulled from borosilicate glass on a PC-10 puller (Narishige, Japan) to a resistance of 5 MΩ for somatic recordings: 7–10 MΩ for dendritic recordings and 5–10 MΩ for axonal recordings. For the olive experiments, the internal solution contained (in mM) 130 KMeSO_4_, 7 KCl, 0.1 EGTA, 2 Na_2_ATP, 2 MgATP, 0.3 Na_2_GTP, 0.5% biocytin, pH 7.3. For Purkinje cell experiments, the internal solution contained (in mM) 133 KMeSO_4_, 7.4 KCl, 0.3 MgCl_2_, 10 HEPES, 0.1 EGTA, 3 Na_2_ATP, 0.3 Na_2_GTP, pH 7.3; except for plasticity experiments, where the internal solution contained 130 K-methanesulfonate, 7 KCl, 0.05 EGTA, 2 Na_2_ATP, 2 MgATP, 0.5 Na_2_GTP, pH 7.3. Patch-clamp recordings were made using a Multiclamp 700A or Axoclamp 2B amplifiers (Axon Instruments, Union City, CA).

Neurons were identified using an upright microscope (BX51; Olympus Optical, Southall, UK or Axioskop; Zeiss, Oberkochen, Germany) under infrared oblique illumination or infrared DIC optics, using either a high-resolution cooled CCD camera (Imago QE; T.I.L.L. Photonics, Martinsried, Germany) or a standard CCD camera (VX-55; T.I.L.L. Photonics). To aid visualization of the axon of olivary neurons, 90 μM Alexa 488 (Molecular Probes, Eugene, OR) was included in the internal solution. Fluorescence excitation was minimized by using brief exposures (80 ms, 2–5 Hz) timed with a monochromator (Polychrome IV; T.I.L.L. Photonics). Cell-attached recordings (seal resistance 50–400 MΩ) were made from the axon and from axon “blebs” forming at the cut ends of axons (mean distance 92 ± 12 μm from the soma) (Khaliq and Raman, 2005; Kole et al., 2007; Monsivais et al., 2005; Shu et al., 2006); whole-cell axonal recordings were made only from blebs. Purkinje cell dendritic whole-cell patch-clamp recordings were made at distances of 85–115 μm from the soma as described previously (Davie et al., 2008). Synaptic input was activated using ACSF-filled patch pipettes.

Long-term plasticity experiments were performed in current-clamp mode without holding current (i.e., allowing spontaneous spiking) in the presence of SR95531 [2-(3-carboxypropyl)-3-amino-6-(4-methoxyphenyl)pyridazinium bromide; 10 μM]. PF synaptic strength was monitored every 10 s at −65 mV during 500 ms hyperpolarizing current pulses using two stimuli separated by 10 ms, measuring the slope of the EPSP in response to the second stimulus as an index of synaptic efficacy. Long-term plasticity induction was carried out (during spontaneous spiking without holding current) using a burst of five PF stimuli at 100 Hz followed by a single or multiple climbing fiber stimuli (40 ms from onset of first PF stimulus), repeated 25 times at 0.5 Hz.

SSE experiments were carried out in current-clamp mode at −65 mV using standard ACSF. PF EPSPs were measured every 2 s using a single PF stimulus. SSE induction was performed using a burst of four to six (one below threshold—defined as the number of PF stimuli causing >20% depression of EPSP amplitude on their own) PF stimuli at 100 Hz together with a single or multiple climbing fiber stimuli (50 ms from onset of the first PF stimulus). Multiple climbing fiber stimuli were delivered as a burst of three or five at 400 Hz. SSE inductions were separated by a recovery period of at least 90 s, and the different induction protocols were interleaved within a recording.

### In Vivo Electrophysiology

Whole-cell patch-clamp recordings from Purkinje cells in vivo were carried out as previously described (Loewenstein et al., 2005). Sprague-Dawley rats (P18–P23) were anesthetized by intraperitoneal injection of a ketamine (56 mg kg^–1^)/xylazine (8 mg kg^–1^) mixture (K-113, Sigma, USA). The level of anesthesia was monitored continuously and additional anesthetic delivered if required. Patch-clamp recordings were made from Purkinje cells in Crus II and vermis using patch pipettes (4–6 MΩ) filled with internal solution containing (in mM) 130 KMeSO_4_, 7 KCl, 10 HEPES, 0.05 EGTA, 2 MgATP, 2 Na_2_ATP, 0.5 Na_2_GTP, pH 7.2. The internal solution contained 5 mM QX-314 unless otherwise indicated.

### Data Acquisition and Analysis

Data were low-pass filtered at 3–10 kHz and acquired at 20–100 kHz using an ITC-18 board (Instrutech, Port Washington, NY) in conjunction with AxoGraph (AxoGraph Scientific, Australia) software. Analysis was carried out using custom-written software for MatLab (MathWorks, Natick, MA) and Igor Pro (Wavemetrics, Lake Oswego, Oregon). All data are reported as mean ± SEM unless otherwise indicated. Differences between groups were tested for statistical significance using Student's t test. For the whole-cell axonal recordings, the attenuation of the ADP and AHP was calculated by dividing the voltage of the ADP and AHP plateau (measured from rest) in the axon by the corresponding voltages measured at the soma and subtracting the resulting number from 1. Propagation of the spikelets in the somatic complex spike down the Purkinje cell axon was predicted using the separatrix function based on spikelet height and interspike interval described previously (Monsivais et al., 2005). In Purkinje cell dendritic recordings, calcium spikes were identified by indentifying events with a steep rise in the voltage trace associated with a discontinuity (or “kink”) in the dendritic voltage, and a decay faster than the EPSP (Edgerton and Reinhart, 2003; Gasparini et al., 2004; Rancz and Hausser, 2006).
