# Supplementary material for: Encoding of Oscillations by Axonal Bursts in Inferior Olive Neurons
Source: Neuron. 2009 May 14;62(3):388–99. doi: 10.1016/j.neuron.2009.03.023 (PMC2777250; doi:10.1016/j.neuron.2009.03.023)
Supplement: Document S1. Supplemental Figures [file mmc1.pdf]

Neuron, Volume 62

Supplemental Data

Encoding of oscillations by axonal bursts in inferior olive neurons

Alexandre Mathy, Sara S.N. Ho, Jenny T. Davie, Ian C. Duguid,
Beverley A. Clark, Michael Häusser

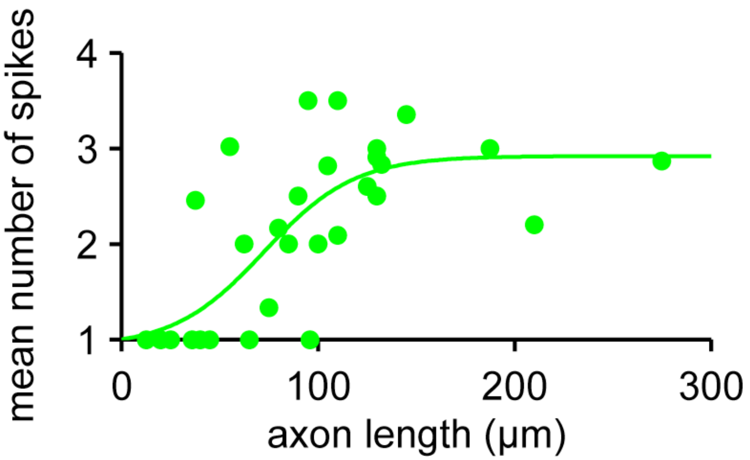

Supplementary Figure 1. Mean number of spikes in an axonal burst as a function of axon length. Same recordings as in Fig. 2B. A sigmoidal fit is shown.

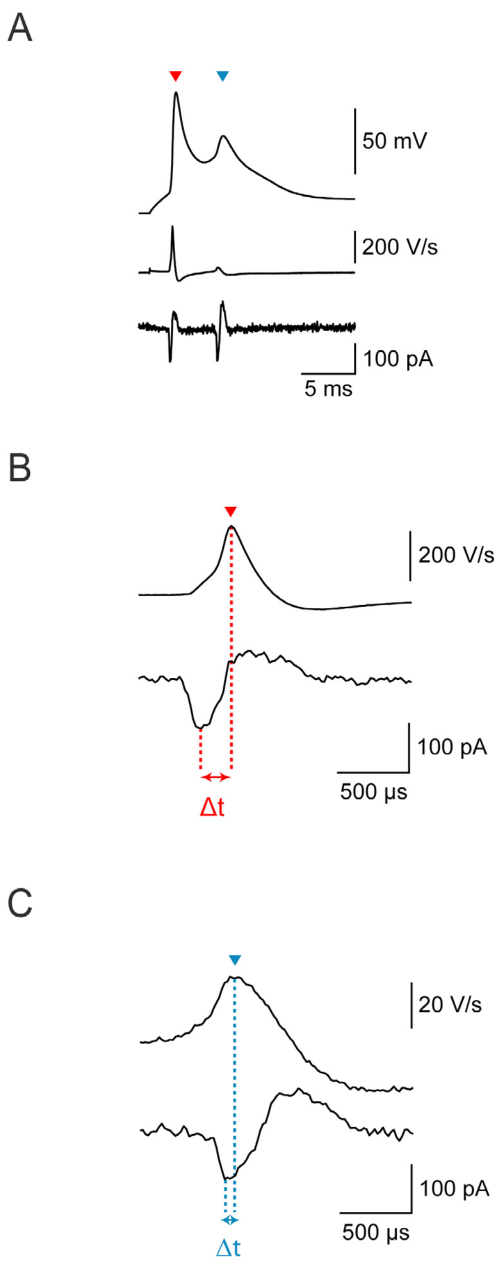

Supplementary Figure 2. Characterization of axonal delays. A) Top trace: somatic recording from an olivary neuron firing a single secondary spike. Middle trace: dV/dt of the somatic trace. Bottom trace: axonal recording (distance 100 µm). B) Top trace: somatic dV/dt of the primary spike at greater magnification. Bottom trace: Axonal recording. The delay is calculated as the time between the occurrence of the peak in the dV/dt and the axonal peak. C) Same as B for the secondary spike.

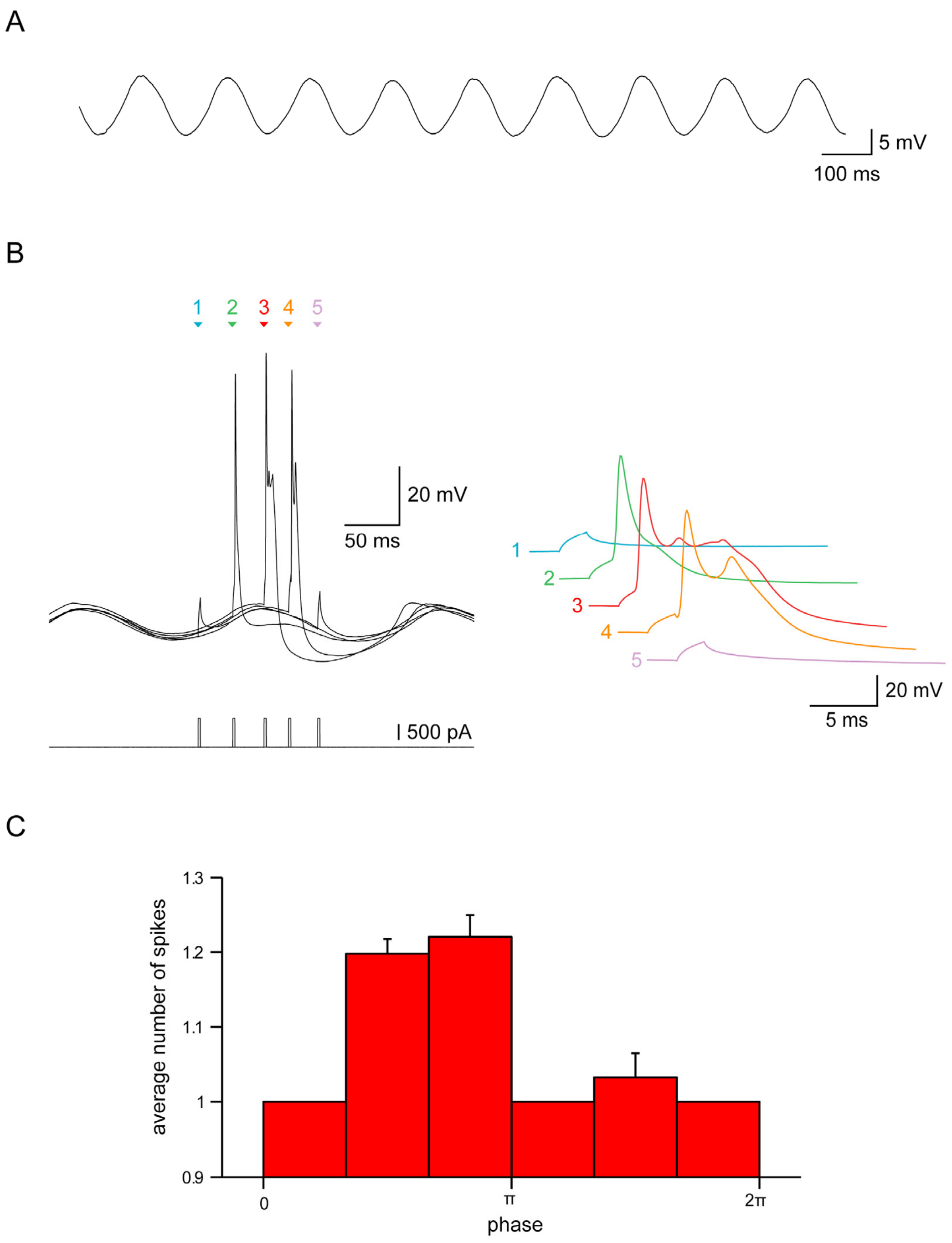

Supplementary Figure 3. Effect of spontaneous subthreshold oscillations on spike output. A) Spontaneous membrane potential oscillation in an olivary neuron. B) Left: current pulses were injected at different phases of the oscillation. Right: Same traces at greater magnification. The number of spikes is greater at the peak of the oscillation. C) Spike output (mean ± SEM) (excluding failures) for different phases of the oscillations (n = 7 cells). A sinusoid function was fit to a short window (50-200ms) of the voltage trace to extract the phase at which the current pulse was injected.

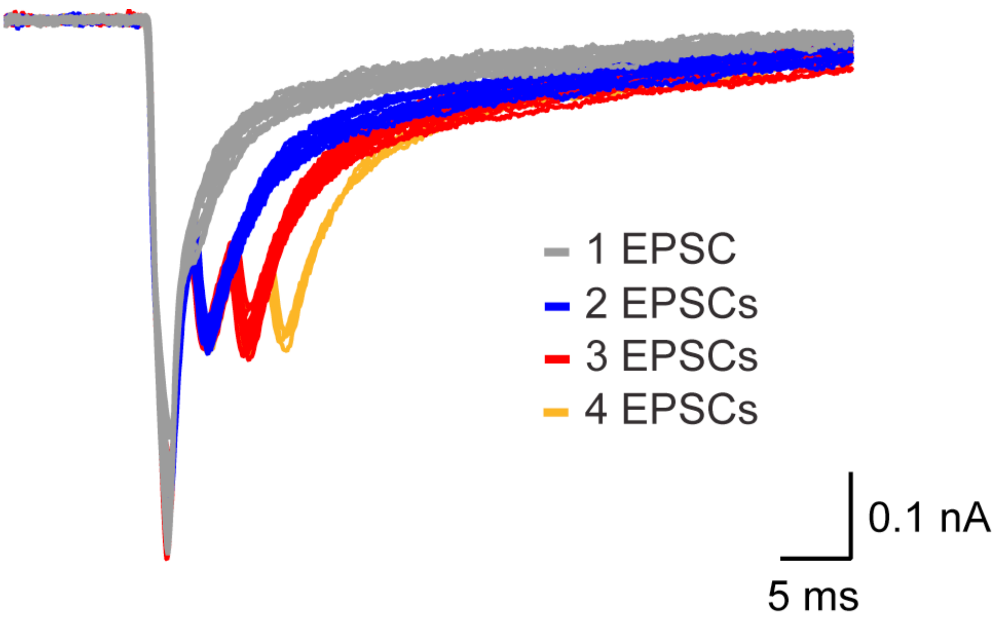

Supplementary Figure 4: Presumed dendritic *in vivo* dendritic recording of a CF EPSC burst. Overlay of 49 successive CF EPSC bursts exhibiting up to 4 individual EPSCs from a presumed Purkinje cell dendritic recording. Voltage clamp holding potential, -100 mV (QX314 was omitted from the pipette solution). The color of each sweep indicates the number of EPSCs in each burst (gray, 1 EPSC; blue, 2 EPSCs; red, 3 EPSCs; orange 4 EPSCs). The EPSC bursts exhibited the following frequency: single EPSC, 33%; double EPSC, 24%; triplet EPSC, 35%; quadruplet EPSC, 8%.

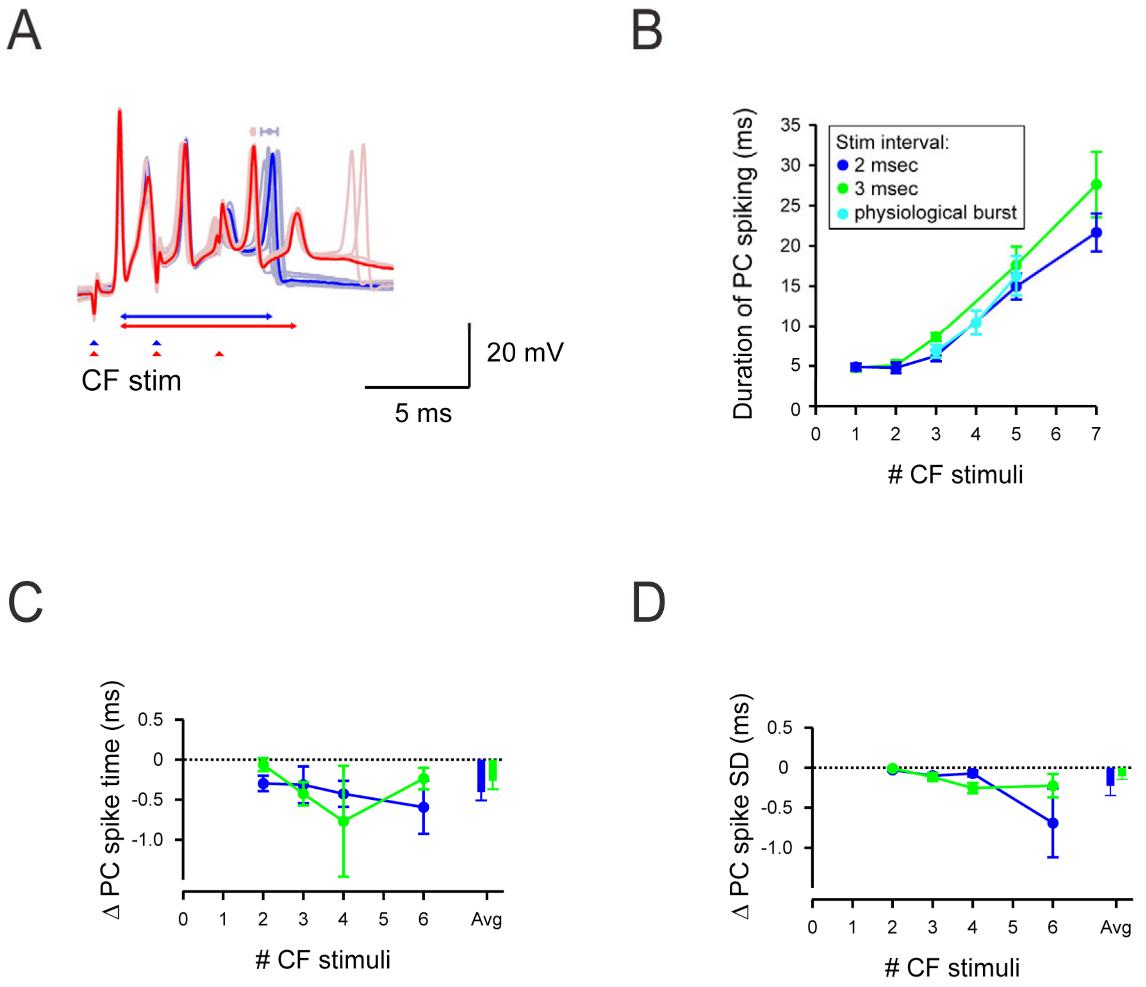

Supplementary Figure 5: Timing of Purkinje cell spikes in response to bursts of CF input. A) Example traces illustrating increase in complex spike duration following addition of a further CF stimulation to the input burst (stimulation given every 3 ms; blue traces, 2 stimuli; red traces, 3 stimuli, 7 traces in each group. A trace is highlighted in both groups). Note the small advance in spike timing and increase in precision of the first spike altered by additional CF stimulation (red and blue dots with error bars above the traces) B) Average (mean ± SEM, n = 8) complex spike duration following bursts of CF input given every 2 (blue) or 3 ms (green), or in physiological patterns (light blue). C) Average (mean ± SEM; n = 8) advance in spike timing and D) increase in spike precision of the first spike altered by addition of a further CF stimulation to the input burst. Colors as in (B).
